# Cymarin

**Published:** 1914-08

**Authors:** 


**Cymarin.** Wiesel, Muench. Med. Woch., page 771, 1914, has used this active principle intramuscularly in doses of 1 m.g. in muscular cardiac insufficiency, collapse, nephritis with uraemia, etc., and speaks favorably of it. In some cases it relieves when digitalis has failed.

